# Diffusion tensor imaging study of brain precentral gyrus and postcentral gyrus during normal brain aging process

**DOI:** 10.1002/brb3.1758

**Published:** 2020-08-25

**Authors:** Ling Zhou, Na Tian, Zuo‐Jun Geng, Bing‐Kun Wu, Li‐Ying Dong, Mei‐Rong Wang

**Affiliations:** ^1^ Department of MRI Xinle City Hospital Xinle Hebei China; ^2^ Department of Infectious Diseases Xinle City Hospital Xinle Hebei China; ^3^ Department of Radiology The Second Hospital of Hebei Medical University Shijiazhuang Hebei China; ^4^ Department of CT The Fifth People's Hospital of Hengshui Hengshui Hebei China

**Keywords:** age‐related analysis, brain imaging, diffusion tensor imaging, fractional anisotropy value, magnetic resonance imaging, postcentral gyrus, precentral gyrus

## Abstract

**Objective:**

To study the changes of white matter tracts in precentral gyrus and postcentral gyrus during normal brain aging process by analyzing fractional anisotropy (FA) values obtained from diffusion tensor imaging (DTI) technology.

**Methods:**

Magnetic resonance imaging (MRI) and diffusion tensor imaging (DTI) were conducted on 120 healthy right‐handed subjects. The subjects were separated into four age groups, namely Young Male/Female (<45 years old) and Senior Male/Female (>45 years old). Each subject undertakes routine MRI and DTI scans. Left/right precentral and left/right postcentral gyrus are automatically detected on the image. The area for region of interest (ROI) is set to be 18 ± 2 mm^2^.

**Results:**

For each age group, the FA values of white matter in precentral gyrus and postcentral gyrus are statistically different (*p* < .05) in both left and right sides of the brain across different age groups and genders. Additionally, the FA values are statistically different (*p* < .05) between two young and senior age groups across different genders, brain regions, and hemispheres.

**Conclusion:**

The FA values of precentral gyrus and postcentral gyrus are statistically different across genders, age groups, and hemispheres. Additionally, the FA values of both precentral gyrus and postcentral gyrus decrease over time, which is a strong indication of aging.

## Graphical abstract

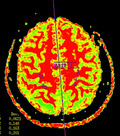

## 1 INTRODUCTION

Diffusion tensor imaging (DTI) is a noninvasive imaging technique based on functional magnetic resonance imaging (MRI) (Basser, Mattiello, & LeBihan, 1994; Jones & Leemans, 2011; Le Bihan, Mangin, & Poupon, 2001). This technology can provide anatomical and functional information, especially for the quantitative evaluation of the integrity of white matter tracts (Assaf & Pasternak, 2008). This is because in brain white matter, the majority of the water molecules diffuse along the axons, the significant anisotropy of the water diffusion is considered to be an ideal probe for studying the shape, density, spatial orientation, and integrity of myelin sheath, microtube, and microfiber (Kochunov, Williamson, & Lancaster, 2012). A full set of parameters could be obtained from DTI studies. These parameters include mean diffusivity (MD), fractional anisotropy (FA), axial diffusivity (AD), and radial diffusivity (RD) (Landman et al., 2007). One of the most commonly utilized parameter is FA (Sorensen, Wu, & Copen, 1999). To be specific, FA values refer to the percentage of water molecules that diffuse along the axon as compared to other directions. The closer FA value is to 1, the more anisotropic the white matter is, and the better the integrity of the white matter is preserved (Büchel et al., 2004). precentral gyrus and postcentral gyrus are the sites where primary motor cortex and primary somatosensory cortex are located, respectively. Although there are numerous reports on the DTI study of white matter (Madden, Bennett, & Song 2009; Madden et al., 2012), the majority of them were focused on diseases models such as ischemic stroke (Kunimatsu et al., 2003), ALS (Bowen, Pattany, & Bradley, 2000), schizophrenia, and (Kubicki, Park, & Westin, 2005) Alzheimer's Disease (Douaud, Jbabdi, & Behrens, 2011). To the best of our knowledge, there is no report on the changes of the microscopic structure and function of precentral gyrus and postcentral gyrus during normal aging process. Given there is an increasingly medical need for the treatment/preventing of motor and sensation impairment among the senior population (Seidler, Bernard, & Burutolu, 2010), the present study is focused on structural changes of precentral gyrus and postcentral gyrus during normal aging process by using DTI as a tool.

## 2 MATERIALS AND METHODS

This study was approved by the Ethics Committee of the Second Hospital of Hebei Medical University. The study was conducted in between March 2015 and December 2015. Healthy subjects (*n* = 120) were separated into four groups according to the guidelines of World Health Organization (WHO): Young Male (*n* = 30) and Female (*n* = 30), with the age ranging from 20 to 44, average age = 35; Senior Male (*n* = 30) and Female (*n* = 30), with the age from 45 to 81, average age = 59.

### 2.1 Inclusion criteria

1. Right‐handed; 2. Mini‐mental State Examination score > 27; 3. no symptoms of neurological diseases; 4. no history of brain surgery or traumatic brain injury, hypertension, diabetes, or cardiovascular diseases; 5. no history of alcohol abuse, smoking addiction, or drug addiction; 6. no MRI contraindication; 7. no history of epilepsy or meningitis; 8. no history of cerebral anoxia or chronic hypoglycemia; and 9. no lesions observed during routine MRI.

### 2.2 Data acquisition

Diffusion tensor imaging was performed on subjects who meet the abovementioned criteria, and the images were further processed to eliminate artifacts and to exclude low‐quality images. All subjects have signed consent forms.

MRI and DTI data were collected on an HDXT 1.5 T scanner (General Electric Health Care) with 8‐channel head coil. The scanning protocol includes axial DTI with echo planar imaging (EPI) sequence: TR/TE = 8000/98.3 ms, slice thickness = 4.0 mm, slice gap = 0.0 mm, FOV = 22 × 22 cm, and NEX = 2; the scan was performed from the lower edge of corpus callosum to the top edge of the brain with a total of 18 slices. In addition to the DTI acquirement, which was used for the analysis here, the scanning protocol included structural sequences to rule out any impairment. The duration of the scan is 4 min 24 s.

### 2.3 Image processing

**FIGURE 1:**
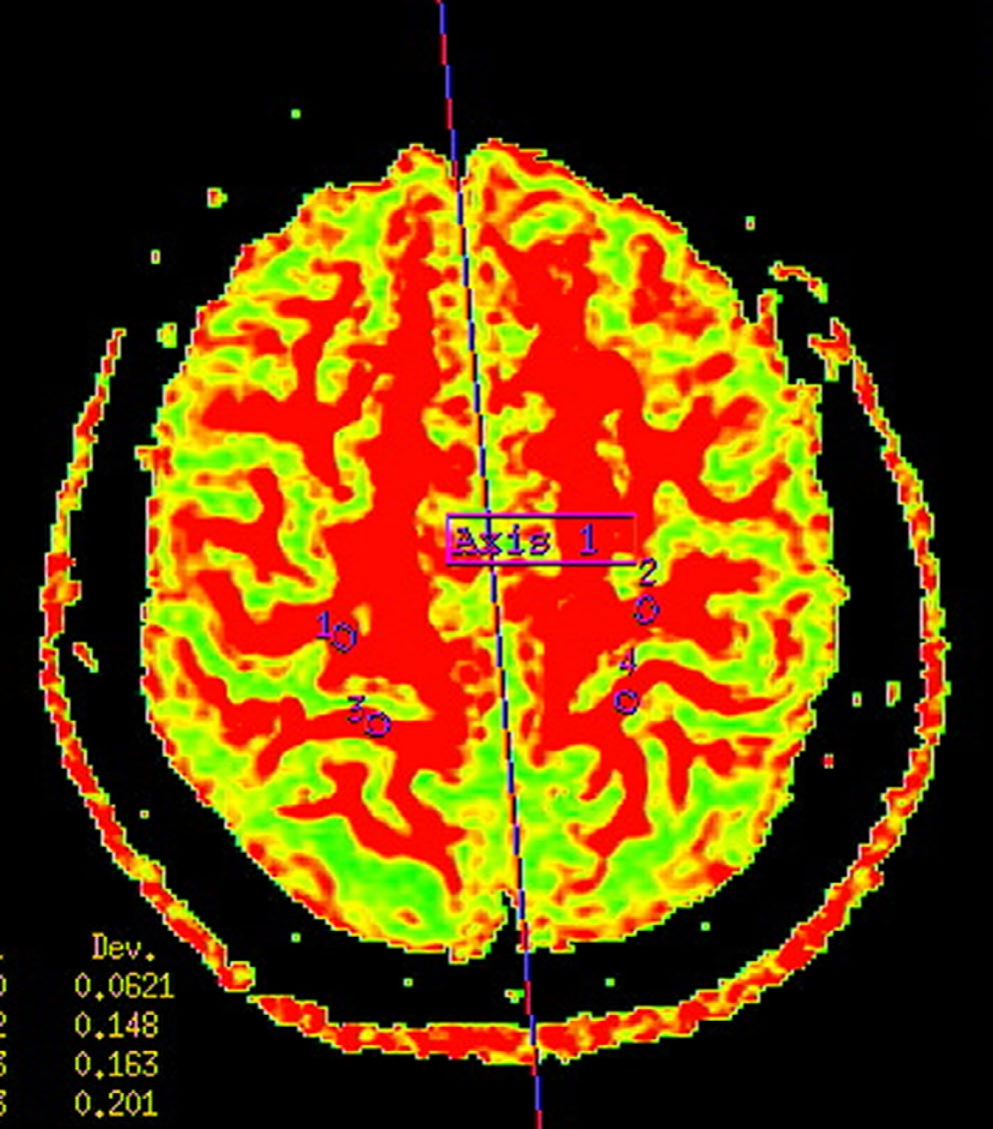
Location of left precentral gyrus, right precentral gyrus, left postcentral gyrus, and right postcentral gyrus (1. right precentral gyrus; 2. right postcentral gyrus; 3. left precentral gyrus; and 4. left postcentral gyrus)

Diffusion tensor imaging data were processed on an ADW 4.5 workstation equipped with Functool software to generate images. The precentral gyrus and postcentral gyrus on one side of the brain are identified by an experienced radiologist, and a region of interest (ROI) with an area of (18 ± 2) mm^2^ was manually selected on this side. Subsequently, by using the auto‐detection function of the program, precentral gyrus and postcentral gyrus on the opposite site of the brain are detected automatically (Figure 1) with the same area of ROI, (18 ± 2) mm^2^. Depending on the size and shape of the precentral gyrus and postcentral gyrus, minor modifications were adopted based on the automated selection of ROI for each specific case in order to avoid the inclusion of encephalocoele or cerebrospinal fluid. To reduce variations and maintain the isotropy of the size of the voxel, FA values were calculated based on the data collected from three slices, namely slices 12–14, and was taken average of 3 measurements.

### 2.4 Statistical analysis

The statistical analysis was conducted with commercial software SPSS 21 for *t* test. All results were shown in average with a standard deviation. *p* value was calculated to determine whether there is significant difference between the two brain regions that are studied (i.e., precentral gyrus and postcentral gyrus).

## 3 RESULTS

The FA values obtained from DTI scanning showed a significant difference between precentral gyrus and postcentral gyrus in all age groups comparing the same side of the brain. The data are summarized in Tables 1 and 2. For example, as shown in Table 1, within Young Female group, on the left side of the brain, there was an average FA value of 0.614 in precentral gyrus as compared to 0.567 in postcentral gyrus. *p* value was determined to be .02 for this age group. Similarly, within Senior Female Group, on the left side of the brain, there was an average FA value of 0.501 in precentral gyrus while an average FA value of 0.445 in the postcentral gyrus. *p* value was determined to be <.001 for this age group. The right side of the brain exhibits similar difference in the FA values between precentral gyrus and postcentral gyrus.

**TABLE 1:** FA values of precentral gyrus and postcentral gyrus for female group

Brain side	Region	Average FA	*T/Z*	*p*
Left side	Young female precentral gyrus	0.614 ± 0.054	*T *= −3.310	.002
	Young female postcentral gyrus	0.567 ± 0.054		
	Senior female precentral gyrus	0.501 ± 0.056	*T *= −4.183	<.001
	Senior female postcentral gyrus	0.445 ± 0.047		
Right side	Young female precentral gyrus	0.646 ± 0.050	*T *= −5.304	<.001
	Young female postcentral gyrus	0.575 ± 0.054		
	Senior female precentral gyrus	0.523 ± 0.057	*Z *= −4.244	<.001
	Senior female postcentral gyrus	0.447 ± 0.054		

**TABLE 2:** FA values of precentral gyrus and postcentral gyrus for male group

Brain side	Region	Average FA	*T/Z*	*p*
Left side	Young male precentral gyrus	0.631 ± 0.044	*Z *= −4.096	<.001
	Young male postcentral gyrus	0.571 ± 0.050		
	Senior male precentral gyrus	0.521 ± 0.066	*Z *= −4.709	<.001
	Senior male postcentral gyrus	0.433 ± 0.060		
Right side	Young male precentral gyrus	0.639 ± 0.044	*T *= −4.907	<.001
	Young male postcentral gyrus	0.572 ± 0.061		
	Senior male precentral gyrus	0.507 ± 0.071	*Z *= −3.630	<.001
	Senior male postcentral gyrus	0.452 ± 0.044		

As summarized in Table 2, within Young Male Group, on the left side, there was an average FA value of 0.631 in precentral gyrus and 0.571 in postcentral gyrus. Similarly, within Senior Male Group, on the left side, there was an average FA value of 0.521 in precentral gyrus and 0.433 in postcentral gyrus. The *p* values were determined to be <.001 in both age groups of male subjects on the left side. The right side of the brain exhibits similar difference in the FA values between pre‐ and postcentral gyrus.

Table 3 demonstrates the difference of FA values across different age groups for females. Specifically, for precentral gyrus on the left side, the Young Group showed an FA value of 0.614 whereas the Senior Group showed an FA value of 0.500. The *p* value was determined to be <.001. For postcentral gyrus on the left side, the Young Group showed an FA value of 0.567 whereas the Senior Group showed an FA value of 0.445. The *p* value was determined to be <.001. The data obtained from the right side showed similar pattern as those on the left side for Young Group and Senior Group.

**TABLE 3:** FA values across different age groups for females

	FA value of precentral gyrus on left side	FA value of postcentral gyrus on left side	FA value of precentral gyrus on right side	FA value of postcentral gyrus on right side
Young	0.614 ± 0.054	0.567 ± 0.054	0.646 ± 0.050	0.575 ± 0.054
Senior	0.500 ± 0.056	0.445 ± 0.047	0.523 ± 0.057	0.447 ± 0.054
*T*	0.319	0.964	0.910	0.006
*p*	<.001	<.001	<.001	<.001

Table 4 demonstrates the difference of FA values across different age groups for males. Specifically, for precentral gyrus on the left side, the Young Group showed an FA value of 0.631 whereas the Senior Group showed an FA value of 0.521. The *p* value was determined to be <.001. For postcentral gyrus on the left side, the Young Group showed an FA value of 0.571 whereas the Senior Group showed an FA value of 0.433. The *p* value was determined to be <.001. The data obtained from the right side showed similar pattern as those on the left side for Young Group and Senior Group.

**TABLE 4:** FA values across different age groups for males

	FA value of precentral gyrus on left side	FA value of postcentral gyrus on left side	FA value of precentral gyrus on right side	FA value of postcentral gyrus on right side
Young	0.631 ± 0.044	0.571 ± 0.050	0.639 ± 0.044	0.572 ± 0.061
Senior	0.521 ± 0.066	0.433 ± 0.060	0.507 ± 0.071	0.452 ± 0.044
*T*	1.788	0.0481	4.471	6.372
*p*	<.001	<.001	<.001	<.001

## 4 DISCUSSION

The significant difference of FA value between the precentral gyrus and postcentral gyrus reflects that their microscopic structures are distinct from each other due to different roles they plan in motion control and body sensation. The distinct functionalities of precentral gyrus and postcentral gyrus are consistent with the observed difference of FA values between these two brain regions for all age groups. As summarized in Tables 1 and 2, the FA values of precentral gyrus from different age groups are invariably greater than those of the postcentral gyrus, further indicating a greater anisotropy character of the white matter that controls the motor function of the body.

Furthermore, as shown in Tables 3 and 4, FA values decrease over time for both females and males. This is a clear indication that the integrity of white matter is gradually sacrificed during aging process. Previous studies have shown that the anterior side of the brain structure, such as corpus callosum and frontal lobe, tends to have an inverse relationship between FA value and age. In contrast, structures on the posterior side such as splenium, parietal lobe, occipital lobe, and internal capsule have relative stable FA values over time (Lebel, Caverhill‐Godkewitsch, & Beaulieu, 2010; Meyer et al., 1996; Michielse, Coupland, & Camicioli, 2010; Sullivan, Adalsteinsson, & Pfefferbaum, 2005). Our study revealed that although FA values follow the trend of decreasing over time, the FA value of precentral gyrus on the posterior side is greater than that of postcentral gyrus on the anterior side. In addition, it was reported that precentral gyrus has a greater volume than the postcentral gyrus, which is called wide belt sign (Damoiseaux, Smith, & Witter, 2009). Because the motor function controlled by precentral gyrus is more adaptable to acquired physical training (Blakemore & Frith, 2005; Pascual‐Leone, Amedi, Fregni, & Merabet, 2005), senior individuals who are exposed to various amount of training related to motor functions can have significant reduction in the rate of aging of precentral gyrus. Wei et al. disclosed that diving athletes have a higher density of gray matter in precentral gyrus and thalamus (Wei, Luo, & Li, 2009). It is possible to delay the onset of brain aging by physical training.

It is worthwhile to note that Male Group (Young and Senior) tends to have slightly higher FA values than Female Group (Young and Senior). The difference across the two genders also possibly arises from the difference in the amount of acquired physical training that helps slow down the rate of brain aging. In addition, as shown in Tables 1 and 2, FA values on the left side of the brain are generally higher than those on the right side. Since all subjects tested are right‐handed, this observation is consistent with the fact that both motor and sensory functions are controlled by the opposite side of the brain.

However, given the preliminary nature of the present study, certain subjects with brain atrophy who does not have observable symptoms might be included in the study as healthy subjects. Moreover, the presence of cerebrospinal fluid could interfere with the measurement of FA values during imaging experiments. Future experiments that address these limitations are well underway in our laboratory.

## 5 CONCLUSION

Our study analyzed the FA values of both brain regions in different age groups using DTI scanning and discovered the microscopic environment in brain white matter changes over time for both precentral gyrus and postcentral gyrus. The decreasing FA values in both precentral gyrus and postcentral gyrus indicate that the integrity of the white matter reduces during the aging process. precentral gyrus and postcentral gyrus are primary motion cortex and primary sensation cortex, respectively. The present study demonstrates that the FA values for precentral gyrus are statistically greater than the FA values of postcentral gyrus, suggesting a greater anisotropy of the white matter that controls the motor function of the body, since the proper function of these two structures has significant impact on the quality of lives of the aging population. The present research provides valuable insights into the search for more effective treatment for motion or sensation related neurological diseases for aging population.

## CONFLICT OF INTEREST

The authors declare there is no conflict of interest.

## AUTHOR CONTRIBUTION

Ling Zhou and Zuo‐Jun Geng contributed to the conception and design of the study. All authors participated in the clinical practice, including diagnosis, treatment, consultation, and follow‐up of patients. Mei‐Rong Wang and Li‐Ying Dong contributed to the acquisition of data. Bing‐Kun Wu and Mei‐Rong Wang contributed to the analysis of data. Ling Zhou wrote the manuscript. Zuo‐Jun Geng revised the manuscript. All authors approved the final version of the manuscript.

### Peer Review

The peer review history for this article is available at https://publons.com/publon/10.1002/brb3.1758.

## Data Availability

The datasets generated and analyzed during the current study are available from the corresponding author on reasonable request.
